# Effects of the Ratio of Insoluble Fiber to Soluble Fiber in Gestation Diets on Sow Performance and Offspring Intestinal Development

**DOI:** 10.3390/ani9070422

**Published:** 2019-07-05

**Authors:** Yang Li, Lijia Zhang, Haoyu Liu, Yi Yang, Jiaqi He, Meng Cao, Min Yang, Wei Zhong, Yan Lin, Yong Zhuo, Zhengfeng Fang, Lianqiang Che, Bin Feng, Shengyu Xu, Jian Li, Xilun Zhao, Xuemei Jiang, De Wu

**Affiliations:** Key Laboratory for Animal Disease-Resistance Nutrition of the Ministry of Education, Animal Nutrition Institute, Sichuan Agricultural University, Chengdu 611130, China

**Keywords:** soluble fiber, insoluble fiber, ratio, sow, small intestine, piglets, performance

## Abstract

**Simple Summary:**

Gestating sows fed a diet rich in dietary fiber show improved performance. Dietary fiber is composed of insoluble fiber and soluble fiber. The ratio of insoluble to soluble fiber may affect overall diet utilization and influence sow performance. Maternal nutrition significantly affects offspring intestinal development; therefore, we investigated the effects of the ratio of insoluble to soluble fiber in gestation diets on sow performance and offspring intestinal development. Our results suggested that, when the dietary fiber levels were the same in gestation diets, the ratio of insoluble to soluble fiber affected the development of intestinal morphology and enzymatic activity related to nutrient digestion and absorption, and consequently affected the average daily gain during lactation and average piglet body weight at weaning. When the ratio of insoluble to soluble fiber was 3.89 in the gestation diet, higher average piglet body weight and litter weight at weaning were observed. These results may provide guidance for the application of fiber in pig production.

**Abstract:**

To investigate the effects of the ratio of insoluble fiber to soluble fiber (ISF:SF) on sow performance and piglet intestinal development, we randomly assigned 64 gilts to four treatments comprising diets with the same level of dietary fiber, but different ISF:SF values of 3.89 (T1), 5.59 (T2), 9.12 (T3), and 12.81 (T4). At birth and weaning, six piglets per treatment at each phase were slaughtered for sampling. As ISF:SF increased, the mean piglet body weight (BW) at weaning and piglet BW gain, which were all significantly higher in T1 and T2 compared with T3 and T4 (*p* < 0.05), showed a linear decrease (*p* < 0.05); the crypt depth of the jejunum in weaned piglets linearly increased, whereas the duodenal weight, jejunal villus height, and villus height/crypt depth in newborn piglets and enzymatic activity of lactase, sucrase, and maltase linearly decreased (*p* < 0.05). No differences were observed in the yield and composition of milk (*p* > 0.05). Moreover, when the ISF:SF was 3.89 in gestation diets, higher piglet BW at weaning occurred, possibly because the ISF:SF affected development and enzymatic activity in the small intestine—effects related to digestion and absorption of nutrients—and consequently enhanced piglet BW gain.

## 1. Introduction

Previous studies have demonstrated that gestating sows fed a diet rich in dietary fiber (DF) show improved performance. Crude fiber (CF), acid detergent fiber (ADF), and neutral detergent fiber (NDF) analyses are primarily used for animal nutrition and the analysis of roughage. DF cannot be digested by the enzymes in the mammalian small intestine, and it is conventionally classified into two categories according to water solubility: insoluble fiber (ISF) such as cellulose and lignin, and soluble fiber (SF) such as pectin, gums, and inulin [1]. Research has described the effects of fiber on the reproductive performance of pregnant sows according to the soluble and insoluble fiber properties, because the water-soluble part is lost during the determination of CF, ADF, and NDF [2]. Guillemet et al. [3] have reported that feeding a gestating sows high-fiber diet containing wheat bran, sugar beet pulp, and soybean hulls to increase the content of both SF and ISF results in improved piglet growth rate and a tendency to increased body weight (BW) at weaning. The addition of ground wheat straw containing large amounts of ISF to the gestation diet also improves sow and litter performance, resulting in increased total litter weight at birth and weaning [4]. Moreover, gestating sows fed a diet rich in inulin, a type of SF, show increased piglet BW at weaning [5]. SF can be easily fermented by the microbiota to produce volatile fatty acids, which are beneficial to the health of the host [6,7,8]; moreover, increased SF intake increases the bacterial population in the large intestine [9]. Insoluble fiber can bind water, thereby increasing fecal volume and promoting regular bowel movements, which may be conductive to decreasing the production and absorption of endotoxins [10]. Therefore, combining ISF and SF in diets may improve indices of gut health. The ratio of ISF to SF (ISF:SF) in fiber affects the overall diet utilization and appears to be important in the formulation of diets. Burkhalter et al. [11] have observed the highest ileal digestibility in dogs with diets with an ISF:SF of 1.9 and 7.2. The ISF:SF in gestation diets in China and America ranges from 7 to 11 because of a corn-soybean meal diet, whereas in Europe, the ratio ranges from 3 to 5 because of a corn-soybean-barley-oat meal diet. Barley and oats both contain abundant SF. However, the optimal ISF:SF for improving sow performance has not been reported. 

The small intestine is major site of digestion and absorption of nutrients. Many studies have demonstrated that high fiber diets promote intestinal development in pigs [12,13]. Recently, the effects of maternal nutrients on the growth and development of offspring have become a growing concern. Our previous research has shown that the maternal nutrition level affects the intestinal morphology and enzymatic activity in newborn and weaned pigs [14]. Cheng et al. [15] have also shown that a maternal SF diet during pregnancy improves growth performance and decreases intestinal permeability in piglets. However, there is little information available in the scientific literature on the effects of maternal ISF:SF intake on intestinal development in offspring. 

In this study, inulin and cellulose were used to adjust the ISF:SF in gestation diets to investigate the effect of ISF:SF with the same level of DF on sow reproductive performance and piglet growth performance and intestinal development.

## 2. Materials and Methods

The present experiment was conducted at the Research Farm of the Animal Nutrition Institute, Sichuan Agricultural University, Ya’an, China. The experimental protocol used in the present study was approved by the Animal Care and Use Committee of Sichuan Agricultural University and followed the current laws of animal protection (Ethics Approval Code: SCAUAC201408-3).

### 2.1. Animals and Diets

A total of 64 Large White × Landrace crossbred gilts with similar BW and backfat thickness (BF) were used in this study. Sows were inseminated artificially 12 h after the first sign of estrus, then again 12 h later with fresh semen obtained from Duroc boars, and were checked for signs of estrus by using a mature boar from day 16 to 21 after insemination. Sows that returned to estrus after insemination were not used in this experiment. After insemination at the fourth estrus, gilts were assigned randomly to four treatments (16 replicates per treatment) comprising diets with the same level of DF, but different ISF:SF of 3.89, 5.59, 9.12, and 12.81, denoted T1, T2, T3, and T4, respectively.

### 2.2. Diets and Management

The four gestation dietary treatments were all a corn-soybean diet (Table 1) differing only in the content of cellulose (Guangxi Shangda Tech Co., Nanning, China) and inulin (ZTH Tech, Beijing, China), which were used to adjust the ISF:SF. Values from National Research Council (NRC, 2012) were used to calculate the chemical composition of the gestation diets. Gilts were fed once daily at9 a.m. The daily gestation diet intake per sow was 2.37 kg from day 1 to 89, 2.86 kg from day 90 to 111, and 1.90 kg from day 112 to parturition for all treatment groups. Two sows were culled because of non-estrus and return to estrus. 

Sows were moved from gestation stalls to farrowing rooms on day 110 of gestation and were kept in individual farrowing crates thereafter. During lactation, all sows were fed ad libitum with the same fortified corn-soybean meal lactation diet (Table 2). All gestation diets and the lactation diet met or exceeded the nutrient requirements for sows according to the NRC (2012). During the 24 h post-farrowing, the litter sizes were adjusted to 8–12 piglets per nest by cross-fostering within the same treatment. All sows consumed a standard lactation diet starting at 2.0 kg/day, then increasing by 0.5 kg⁄day during the first 5 days after parturition, and they were given ad libitum access to the diet thereafter. Sow’s milk was the unique source of food to the suckling piglet during lactation. All sows and piglets were given free access to water throughout the experiment through nipple drinkers. All piglets were weaned at day 28 of lactation. The temperature was maintained at 22–26 °C in the farrowing house and gestation stalls, and heat lamps provided supplemental heat to the piglets. 

### 2.3. Measurements

Sows were weighed individually at breeding, day 110 of gestation, within 24 h of farrowing and weaning. The BF thicknesses at the last rib were measured at breeding, day 110 of gestation, and weaning with a Digital Diagnostic Ultrasound Devices (HG 9300, Caresono Technology Co., Ltd., Nanjing, China). The numbers and BW of the live pigs born per litter at farrowing, and the number of live pigs per litter and weaning-to-estrus interval at weaning were recorded for each sow and litter. Daily feed intakes were recorded for each sow during lactation.

The milk yield in this experiment was estimated with the weigh-suckle-weigh method described by Speer and Cox [16] and Špinka et al. [17] on days 2 and 15 between 9 a.m. and 4 p.m. after the day of farrowing. Pigs were removed from the sow in the morning and placed in a cubicle that provided supplemental heat when required. Equal amounts of weighed daily diets were given to the sows. Approximately 55 min later, the pigs were moved to a cold and damp concrete floor, which encouraged the pigs to urinate and defecate. The pigs were weighed for the first time (*W_1_*) after two or more pigs started activity or the sow lay on her side, exposing the udder, and began grunting. The litter was released into the pen after the last pig was weighed. When the first pig moved away, all pigs were immediately gathered and weighed a second time (*W_2_*), and the nursing time (*t*) was recorded. The procedure was repeated at hourly intervals for eight consecutive hours. The milk intake of each pig was then based on the weight difference before and after the suckling, and was corrected for metabolic losses between the weighings, as described by Noblet and Etienne [18]. The milk intake *M* was calculated as: *M* = *W_2_* − *W_1_* + (0.21*t*)/(*W*_1_^0.75^). The hourly milk yield for the sow was obtained by summing daily milk intake and dividing the total nursing time for the seven procedures. In all cases, when a pig was observed to urinate or defecate during the nursing period, a correction of 10 and 5 g, respectively, in the weight gain of the litter was made, as proposed by Legagnuer [19].

### 2.4. Sampling Procedure

Colostrum samples (15 mL) from eight sows per treatment were collected by hand within 2 h after the birth of the first piglet, and milk samples (15 mL) were collected from all functional glands at day 14 of lactation after injection of 2 mL of oxytocin (20 USP units/mL) into the ear vein, as described by Shen et al. [20]. The colostrum and milk samples were refrigerated at −20 °C immediately before analysis of composition with a Rapid Milk Analyzer (MILKYWAY-CP2, Beijing KANGGAOTE Science and Technology Co., Ltd., Beijing, China)

At birth, a total of 24 piglets from different litters, six per treatment, with a BW closest to the average weight in each treatment, were slaughtered without consuming colostrum, and the remaining piglets were kept with the sows. Similarly, a total of 24 piglets from a different litter, six per treatment, were slaughtered after weaning. After evisceration, the small intestine was dissected and rapidly measured for weight and length. The small intestine in piglets was defined as described in Li et al. [21]. The portion of the digestive tract between the pylorus and the ileocecal junction and intestinal segments (duodenum, jejunum and ileum) were obtained by using the anatomical landmarks, with the pyloric-duodenal junction to the duodenal-jejunal junction being the duodenum, the duodenal-jejunal junction to the jejunal-ileal junction being the jejunum, and the jejunal-ileal junction to the ileocecal junction being the ileum. The intestinal index (weight/length) was calculated as the intestinal weight divided by the intestinal length, and the relative weight (intestinal weight/BW) was calculated as the intestinal weight divided by the BW. 

### 2.5. Mucosal Morphology Measurements 

After the intestinal segments (duodenum, jejunum, and ileum) were fixed in 4% paraformaldehyde for 24 h, dehydration, clearing, and paraffin embedding were performed. Then serial sections of 5 μm thickness were made, and this was followed by hematoxylin and eosin staining. Two transverse sections of each intestinal sample (duodenum, jejunum, or ileum) were prepared on one slide for morphometric analysis. A total of 12–20 intact, well-oriented crypt-villus units per sample were chosen randomly and measured. Villus height measurements were taken from the tip to the base of the villus between individual villus, and crypt depth was measured from the valley between individual villi to the basal membrane. The small intestinal crypt depth (μm) and villus height (μm) were measured with JD801 morphologic image analysis software (JEDA Science-Technology Development Co., Ltd., Nanjing, China), and then villus height/crypt depth (V/C) was calculated as the villus height divided by the crypt depth.

### 2.6. Enzyme Activity

Disaccharidase activity was determined in the jejunum mucosa. After thawing, 0.5 g of mucosal scraping was homogenized with ice-cold physiological saline and centrifuged for 15 min at 3000× g at 4 °C. Total protein content was determined according to the Bradford method. The activity of lactase, maltase, and sucrase was measured with kits according to the manufacturer’s instructions (Jiancheng Bioengineering Ltd, Nanjing, China).

### 2.7. Statistical Analyses

Sows and their litters were regarded as the experimental units to evaluate performance, and individual piglet data were considered as the experimental units to evaluate other variables by using the General Linear Model (GLM) in SAS (9.0 Inst., Inc., Cary, NC, USA). Variations among the four treatments were compared with Duncan’s multiple comparison test. Preplanned single degree of freedom comparisons were also made to measure the linear and quadratic effects of ISF:SF. Normality of the data was assessed with the Shapiro–Wilk statistic (*W* > 0.05). If the data did not follow a normal distribution, transformation was used to achieve normality of the data. Values are expressed as means ± standard error of the mean. Differences between treatments were considered significant when *p* < 0.05, and a tendency was recognized when 0.05 < *p* < 0.10.

## 3. Results

### 3.1. Reproductive Performance of Sows

The effects of ISF:SF in gestation diets on sow performance are shown in Table 3. The litter BW and average piglet BW at weaning in T1 and T2 were significantly higher than those in T3 and T4 (*p* = 0.010), and linearly decreased as ISF:SF increased (*p* < 0.05). No significant differences were observed in the other indicators (*p* > 0.05).

### 3.2. Growth Performance of Piglets

As shown in Table 4, the BW gains in T1 and T2 from d 0 to 14 and from d 0 to 28 were significantly higher than those in T3 and T4 (*p* < 0.05); that in T1 from d 15 to 28 was markedly higher than those in T3 and T4; and that in T2 was significantly higher than that in T3 (*p* < 0.05). The BW gain of piglets during lactation significantly decreased with increasing ISF:SF (linear, *p* < 0.05; quadratic, *p* < 0.05). 

### 3.3. Yield and Composition of Milk

**Figure 1:**
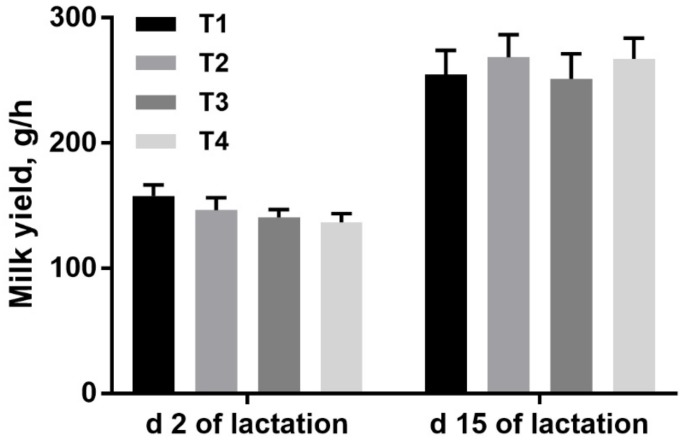
Effects of the ratio of insoluble fiber to soluble fiber in gestation diets on sow milk yield on day 2 and 15 of lactation. No treatment effects and linear and quadratic effects were observed in milk yield on d 2 and 15 of lactation. T1, T2, T3, and T4 were diets in which the ratios of insoluble to soluble fiber were 3.89, 5.59, 9.12, and 12.81, respectively. Values are mean ± standard error. *n* = 16, 15, 15, 16 for T1, T2, T3, and T4, respectively.

No remarkable differences were observed in the yield and composition of milk, as shown in Figure 1 and Table 5, respectively (*p* > 0.05).

### 3.4. Intestinal Development

The effects of ISF:SF in sow gestation diets on the intestinal development of neonatal and weaned piglets are shown in Table 6 and Table 7, respectively. The duodenal weight of neonatal piglets in T1 was significantly higher than that in T4 (*p* = 0.033). As ISF:SF increased, duodenal weight (linear, *p* = 0.002; quadratic, *p* = 0.005) decreased in newborn piglets. There were no significant differences in the measured parameters in weaned piglets (*p* > 0.05). 

### 3.5. Intestinal Morphology

The results of morphometric measurements in the duodenum, jejunum, and ileum in neonatal and weaned piglets are shown in Table 8 and Table 9. For neonatal piglets, the duodenal V/C in T3 was significantly higher than that in T4 (*p* = 0.049) and showed a quadratic tendency (*p* = 0.089). The highest jejunal villus height was observed in T2 and was significantly higher than that in T4 (*p* = 0.037). Compared with T3 and T4, T1 and T2 had markedly higher jejunal V/C (*p* = 0.006). The jejunal villus height showed a linear decrease (*p* = 0.032), and V/C displayed linear (*p* = 0.006) and quadratic (*p* = 0.024) decreases. A markedly lower jejunal crypt depth in weaned piglets was observed in T1 and T2 compared with T3 (*p* = 0.045), and increasing ISF:SF increased the crypt depth of the jejunum (linear, *p* = 0.017). 

### 3.6. Enzyme Activity

The effects of ISF:SF in sow gestation diets on enzymatic activity in the jejunum in neonatal and weaned piglets are shown in Table 10. The lactase activity in newborn piglets and the sucrase and maltase activity in weaned piglets in T3 and T4 were significantly lower than those in in T1 (*p* < 0.05), and those in T4 were markedly lower than those in T2 (*p* = 0.006). The activity of sucrase in newborns and of lactase in weaned piglets in T4 was significantly lower than that under other treatments (*p* < 0.05). However, the activity of maltase of neonatal piglets indicated a linear decrease (*p* < 0.05), and the activity of the other enzymes all showed linear or quadratic decreases with increasing ISF:SF (*p* < 0.05).

## 4. Discussion

In our study, we explored the effects of ISF:SF on sow reproductive performance and piglet growth performance. Significantly higher piglet weaned BW was observed when the ISF:SF was 3.89 or 5.59. Added fiber in gestation diets resulted in inconsistent and inconclusive results based on sow and litter performance when daily energy intake per sow was equalized among treatments. Many studies have shown that fiber supplementation in sow gestation diet increases litter size and piglet BW at birth and weaning and sow feed intake during lactation [2,3,4,22]. However, other studies have found that fiber intake has either no effect or a negative effect on sow performance. The addition of 20% or 40% soybean hulls containing higher ISF to a gestation diet has not been found to improve sow performance when energy intake is equalized among treatments [23,24]. Moreover, the addition of sugar beet pulp containing higher SF to the gestation diet, in a range from 25% to 50% also has not been found to improve sow performance when daily energy intake is equalized among treatments [25,26,27]. The fiber and the ISF:SF may strongly affect sow performance. We observed increased piglet BW at weaning and piglet weight gain during lactation with decreasing ISF:SF. Piglets from sows fed diets supplied with 2% pregelatinized waxy maize starch plus guar gum had higher average daily gain and weaning BW [15]. The non-differential litter size and piglet BW at birth might have been due to the equalized energy intake among the treatments during gestation. However, Vestergaard and Danielsen [27] reported that a diet with high content of SF (50% sugar-beet pulp) had a negative effect on piglet weight at birth. The reasons for the difference might be a result of different ISF:SF in the diets and fiber components in the ingredient. The ISF:SF in their diet was nearly 1:1 which was lower than in ours. It was proved that the ISF:SF in diets had an great effect on the nutrient digestibility [11]. Besides, soluble fiber components in the sugar-beet pulp is pectin. Pectin is a viscous fiber while inulin is a non-viscous fiber [10]. Highly viscous fibers increase the viscosity of the digesta and decrease the digestion and absorption [2,11]. The ISF:SF did not significantly affect sow BW during lactation, possibly because of the similar feed intake during the lactation among all the treatments. Feed intake during lactation is positively correlated with insulin sensitivity in late gestation and early lactation [28]. Both ISF and SF have been shown to improve insulin sensitivity [9,29]. No significant difference was observed in mean BW at weaning between the sows fed sugar-beet pulp diet and wheat bran and oat hulls diet [27]. Milk yield and composition are important factors beneficial to the growth of piglets. However, the ISF:SF had no effect on milk yield and composition. Milk protein content generally is not affected by diet [30]. Krogh et al. [31] have compared the effects of sugar beet pulp and alfalfa meal on the yield and composition of milk in sows and also have found that milk yield is unaffected by dietary fiber. However, the milk protein of the sows fed alfalfa meal was lower than that of the sows fed sugar beet pulp on day 3 and 10 of lactation, possibly because of greater milk yield and lower milk protein. It was also reported that dietary fiber level during gestation did not affect total solids and lactose content of colostrum or milk [32]. Total solids is the sum of solids-not-fat and fat content. Total solids content is highest during the initial 4 to 6 h after parturition, and lactose is present in lower concentrations in colostrum than in mature milk [30]. Consistently, higher total solids and lactose were observed on day 0 of lactation in our study.

Maternal nutrition has been reported to affect intestinal development [13,33]. The small intestine is the main site of absorption of nutrients. The morphology of the intestine is an important indicator of the health of the intestine [34,35]. The villus height determines the ability of the small intestine to absorb nutrients, and the V/C is considered a useful criterion to estimate the nutrient absorption capacity of the small intestine [36]. As ISF:SF increased, the villus weight and V/C in the jejunum in neonatal piglets decreased, and the crypt depth of the jejunum in weaned piglets increased. Previous research has also shown that maternal soluble fiber diets during gestation change the intestinal microbiota, improve growth performance and decrease intestinal permeability in piglets [15]. Moreover, increasing SF intake during gestation would enhance the absorption function of the small intestine in offspring. A similar report has shown that pigs fed diets with added inulin, compared with cellulose, show higher villus height and V/C of the jejunum [37]. Soluble fiber is easily fermented by the gut microbiota, and it increases the bacterial population in the large intestine. The addition of inulin to a gestation diet can not only modulate the intestinal microbiota of sows, but also of their offspring [38]. Maternal gut microbiota passed to offspring might be directly through the lymph/blood circulation during gestation or might reach the mammary gland and then be passed on to offspring during lactation [39] and consequently affect the development of the small intestine [40]. 

We also found that maternal SF intake increased the jejunal relative weight, an important indicator of organ development that tends to increase the intestinal index of neonatal piglets. The increased intestinal index might suggest an enhanced jejunal wall or mucosal thickness. In addition, with increasing ISF:SF, the enzymatic activity of lactase, sucrase, and maltase decreased. Lactase, sucrase, and maltase are important enzymes for the digestion and absorption of colostrum and milk, and lactose is the main carbohydrate in the milk of most placental mammals. Lactase activity is high for the first 2 weeks of life, and then a sharp decline results in minimal levels after 3–4 weeks. In contrast, sucrase and maltase activity increase from inappreciable levels at birth to a maximum after approximately 25 days [41], in agreement with our results. The decrease in enzyme activity is disadvantageous for the digestion of different carbohydrates as sources of energy for growing animals. 

Our research showed that ISF:SF in the gestation diet strongly affected piglet growth performance and intestinal development and enzyme activity, and greater SF in gestation diets was more beneficial than greater ISF. However, exceeded SF intake might adversely affect sow and piglet performance [27]. First, SF could increase the viscosity of the digesta, which might decrease the digestion and absorption [42]. Second, SF can be fermented by gut microbiota and produce a large amount of gas, which would influence colon function, including flatulence and regularity [43]. Third, excessive SF in the diet might increase the risk of metabolic disease [44]. These findings might explain why no significant difference was observed when the ISF:SF was 3.89 and 5.59.

## 5. Conclusions

In conclusion, when the DF levels are the same in the gestation diets, an optimal ISF:SF may improve piglet BW gain through affecting the development of the intestinal morphology and enzyme activity, and consequently enhance the average piglet BW at weaning. Higher average piglet BW at weaning were obtained when ISF:SF was 3.89 in gestation diets. 

## Figures and Tables

**Table 1:** Composition of the experimental diets during pregnancy (as-fed basis).

Items	Treatments ^4^			
	T1	T2	T3	T4
Ingredient, %				
Corn	71.50	71.50	71.50	71.50
Dehulled soybean meal	10.20	10.20	10.20	10.20
Wheat bran	13.00	13.00	13.00	13.00
Cellulose	-	1.00	2.00	2.50
Inulin	2.50	1.50	0.50	-
L-lysine HCl	0.17	0.17	0.17	0.17
DL-methionine	0.01	0.01	0.01	0.01
L-threonine	0.04	0.04	0.04	0.04
Calcium carbonate	0.97	0.97	0.97	0.97
Dicalcium phosphate	0.52	0.52	0.52	0.52
Salt	0.40	0.40	0.40	0.40
Choline chloride	0.14	0.14	0.14	0.14
Vitamin premix ^1^	0.05	0.05	0.05	0.05
Mineral premix ^2^	0.50	0.50	0.50	0.50
Total	100.00	100.00	100.00	100.00
Calculated analysis ^3^				
DE, Mcal/kg	3.05	3.05	3.05	3.05
CP, %	12.13	12.13	12.13	12.13
EE, %	3.22	3.22	3.22	3.22
CF, %	4.83	4.83	4.83	4.83
SF, %	3.87	2.87	1.87	1.37
ISF, %	15.04	16.04	17.04	17.54
ISF:SF	3.89	5.59	9.12	12.81
DF, %	18.91	18.91	18.91	18.91
Ca, %	0.55	0.55	0.55	0.55
AP, %	0.24	0.24	0.24	0.24
Lys, %	0.55	0.55	0.55	0.55
Met, %	0.18	0.18	0.18	0.18
Thr, %	0.41	0.41	0.41	0.41
Trp, %	0.12	0.12	0.12	0.12

^1^ Provided per kg of diet: vitamin A 7500 IU, vitamin D3 5000 IU, vitamin E 37.5 IU, vitamin K3 5 mg, vitamin B1 5 mg, vitamin B2 12.5 mg, vitamin B6 7.5 mg, vitamin B12 0.05 mg, Biotin 0.2 mg, Niacin 50 mg, Folic acid 2.5 mg, D- calcium pantothenate 25 mg. ^2^ Provided per kg of diet: Cu 10 mg (CuSO_4_), Fe 100 mg (FeSO_4_), I 0.6 mg (KI), Zn 100 mg (ZnSO_4_), Mn 30 mg (MnSO4), Se 0.25 mg (Na_2_SeO_3_). ^3^ All data were calculated according to the tables of Feed Composition and Nutrient Values in China (2016) in four diets. ^4^ T1, T2, T3, and T4 were diets in which the ratios of insoluble to soluble fiber were 3.89, 5.59, 9.12, and 12.81, respectively.

**Table 2:** Composition and calculated analysis of lactation diet (as-fed basis).

Item	Lactation
Ingredient, %	
Corn	62.53
Soybean meal	22.74
Wheat bran	5.50
Fish meal	3.00
Soybean oil	2.50
L-lysine HCl	0.23
L-threonine	0.03
L-tryptophan	0.01
Calcium carbonate	1.10
Dicalcium phosphate	1.27
Salt	0.40
Choline chloride	0.14
Vitamin premix ^1^	0.05
Mineral premix ^2^	0.50
Total	100.00
Calculated analysis ^3^	
DE, Mcal/kg	3.31
CP, %	17.55
EE, %	5.61
CF, %	2.67
Ca, %	0.90
AP, %	0.45
Lys, %	1.11
Met, %	0.25
Thr, %	0.58
Trp, %	0.19

^1^ Provided per kg of diet: vitamin A 7500 IU, vitamin D3 5000 IU, vitamin E 37.5 IU, vitamin K3 5 mg, vitamin B1 5 mg, vitamin B2 12.5 mg, vitamin B6 7.5 mg, vitamin B12 0.05 mg, Biotin 0.2 mg, Niacin 50 mg, Folic acid 2.5 mg, D-calcium pantothenate 25 mg. ^2^ Provided per kg of diet: Cu 10 mg (CuSO4), Fe 100 mg (FeSO4), I 0.6 mg (KI), Zn 100 mg (ZnSO_4_), Mn 30 mg (MnSO4), Se 0.25 mg (Na_2_SeO_3_). ^3^ All data were calculated according to the tables of Feed Composition and Nutrient Values in China (2016) in the diet.

**Table 3:** Effects of the ratio of insoluble to soluble fiber in gestation diets on sow performance.

Items ^4^	Treatments ^1^				*p*-Values		
	T1	T2	T3	T4	Treatment	Lin ^3^	Quad ^3^
No. of sows	16	15	15	16			
Sow BW ^2^, kg							
Breeding	153.39 ± 1.66	151.67 ± 2.44	155.05 ± 2.62	153.93 ± 1.87	0.745	0.604	0.861
Gestation, d 110	228.43 ± 2.31	226.65 ± 3.18	225.63 ± 3.97	228.62 ± 2.07	0.922	0.946	0.713
Farrowing	207.85 ± 2.40	203.04 ± 3.24	202.00 ± 2.33	205.43 ± 2.26	0.425	0.531	0.244
Weaning	178.89 ± 3.02	176.32 ± 3.88	180.08 ± 2.66	184.73 ± 3.01	0.300	0.129	0.158
Sow BF ^2^, mm							
Breeding	15.43 ± 0.68	15.85 ± 0.59	15.58 ± 0.80	15.71 ± 0.62	0.961	0.836	0.953
Gestation, d 110	19.25 ± 1.07	18.96 ± 0.73	18.67 ± 0.84	19.31 ± 0.93	0.959	0.834	0.911
Farrowing	18.88 ± 0.75	18.83 ± 0.78	19.36 ± 0.83	19.39 ± 1.25	0.958	0.616	0.881
Weaning	15.00 ± 0.76	14.20 ± 0.68	14.93 ± 0.94	14.83 ± 0.65	0.864	0.930	0.878
Feed intakes during lactation, kg	120.95 ± 2.62	116.76 ± 2.17	106.02 ± 4.35	113.75 ± 7.01	0.175	0.143	0.178
Weaning to estrus interval, d	5.17 ± 0.38	5.10 ± 0.29	4.94 ± 0.20	5.17 ± 0.27	0.948	0.956	0.888
Litter performance							
Pig total born, No./litter	14.00 ± 0.80	15.00 ± 0.59	13.42 ± 0.53	12.62 ± 1.37	0.312	0.438	0.655
Pig born alive, No./litter	11.92 ± 0.70	12.83 ± 0.80	12.00 ± 0.59	11.15 ± 1.22	0.607	0.920	0.851
Average BW ^2^, total pigs at birth, kg	1.46 ± 0.09	1.25 ± 0.05	1.27 ± 0.05	1.25 ± 0.11	0.232	0.318	0.055
Average BW, live pigs at birth, kg	1.40 ± 0.05	1.32 ± 0.05	1.33 ± 0.04	1.28 ± 0.12	0.513	0.560	0.210
Pigs after cross-foster No./litter	10.88 ± 0.30	10.90 ± 0.53	10.43 ± 0.57	10.89 ± 0.31	0.872	0.857	0.903
Average pig BW ^2^ after cross-foster, kg	1.40 ± 0.05	1.36 ± 0.04	1.37 ± 0.05	1.35 ± 0.04	0.840	0.433	0.724
Pigs weaned, No./litter	10.00 ± 0.38	10.10 ± 0.53	9.57 ± 0.75	9.56 ± 0.44	0.827	0.418	0.714
Average pig BW ^2^ at weaning, kg	7.88 ± 0.12 ^a^	7.46 ± 0.15 ^a^	6.80 ± 0.18 ^b^	6.95 ± 0.18 ^b^	<0.001	0.044	0.120

^1^ T1, T2, T3, and T4 were diets in which the ratios of insoluble to soluble fiber were 3.89, 5.59, 9.12, and 12.81, respectively. ^2^ BW, body weight; BF, backfat thickness. ^3^ Lin and quad, linear and quadratic effect of different ratios of insoluble to soluble fiber in pregnant sow diets. ^4^ Values are mean ± standard error. ^a,b^ Means with different superscripts within a row differ (*p* < 0.05).

**Table 4:** Effects of the ratio of insoluble to soluble fiber in gestation diets on piglet weight gain during lactation.

Items ^3^, kg	Treatments ^1^				*p*-Values		
	T1	T2	T3	T4	Treatment	Lin ^2^	Quad ^2^
d 0–14	3.01 ± 0.07 ^a^	2.78 ± 0.08 ^a^	2.52 ± 0.10 ^b^	2.42 ± 0.09 ^b^	<0.001	<0.001	<0.001
d 15–28	3.47 ± 0.06 ^a^	3.33 ± 0.06 ^ab^	2.89 ± 0.11 ^c^	3.18 ± 0.10 ^bc^	<0.001	0.037	0.002
d 0–28	6.48 ± 0.11 ^a^	6.11 ± 0.13 ^a^	5.41 ± 0.18 ^b^	5.61 ± 0.17 ^b^	<0.001	<0.001	<0.001

^1^ T1, T2, T3, and T4 were diets in which the ratios of insoluble to soluble fiber were 3.89, 5.59, 9.12, and 12.81, respectively. ^2^ Lin and quad, linear and quadratic effect of different ratios of insoluble to soluble fiber in pregnant sow diets. ^3^ Values are mean ± standard error. ^a–c^ Means with different superscripts within a row differ (*p* < 0.05). *n* = 16, 15, 15, 16 for T1, T2, T3, and T4, respectively.

**Table 5:** Effects of the ratio of insoluble to soluble fiber in gestation diets on the composition of colostrum and milk.

Items ^3^	Treatments ^1^				*p*-Values		
	T1	T2	T3	T4	Treatment	Lin ^2^	Quad ^2^
Colostrum, %							
Fat	3.43 ± 0.23	3.97 ± 0.0.39	3.32 ± 0.32	3.44 ± 0.30	0.498	0.367	0.525
Solids-not-fat	19.14 ± 0.56	17.74 ± 0.76	17.30 ± 0.41	19.30 ± 0.80	0.120	0.547	0.172
Protein	7.16 ± 0.20	6.54 ± 0.28	6.40 ± 0.15	7.05 ± 0.37	0.214	0.353	0.229
Lactose	5.01 ± 0.10	4.78 ± 0.20	4.66 ± 0.11	4.85 ± 0.18	0.491	0.423	0.303
Milk, %							
Fat	7.29 ± 0.43	7.59 ± 0.70	7.30 ± 0.75	7.95 ± 0.52	0.874	0.577	0.814
Solids-not-fat	10.01 ± 0.11	9.69 ± 0.13	10.19 ± 0.07	9.49 ± 0.13	0.492	0.556	0.691
Protein	3.74 ± 0.04	3.62 ± 0.05	3.81 ± 0.02	3.57 ± 0.07	0.520	0.616	0.747
Lactose	5.34 ± 0.06	5.23 ± 0.06	5.44 ± 0.04	5.01 ± 0.14	0.532	0.471	0.558

^1^ T1, T2, T3, and T4 were diets in which the ratios of insoluble to soluble fiber were 3.89, 5.59, 9.12, and 12.81, respectively. ^2^ Lin and quad, linear and quadratic effect of different ratios of insoluble to soluble fiber in pregnant sow diets. ^3^ Values are mean ± standard error (*n* = 8).

**Table 6:** Effects of the ratio of insoluble to soluble fiber in sow gestation diets on the intestinal development of neonatal piglets.

Items ^4^	Treatments ^1^				*p*-Values		
	T1	T2	T3	T4	Treatment	Lin ^2^	Quad ^2^
Duodenum							
Weight, g	0.54 ± 0.02 ^a^	0.50 ± 0.01 ^ab^	0.48 ± 0.03 ^ab^	0.40 ± 0.10 ^b^	0.033	0.002	0.005
Length, cm	5.25 ± 0.45	5.77 ± 0.67	5.650 ± 0.45	5.217 ± 0.41	0.822	0.487	0.621
Relative weight ^3^, g/kg BW ^2^	0.04 ± 0.01	0.04 ± 0.01	0.04 ± 0.01	0.03 ± 0.01	0.336	0.149	0.212
Intestinal index ^3^, g/cm	0.11 ± 0.01	0.10 ± 0.01	0.09 ± 0.01	0.09 ± 0.01	0.179	0.056	0.113
Jejunum							
Weight, g	31.90 ± 1.79	29.52 ± 2.04	29.80 ± 2.55	30.20 ± 1.93	0.977	0.436	0.693
Length, cm	316.73 ± 14.70	320.52 ± 9.40	331.83 ± 16.16	327.57 ± 15.63	0.875	0.109	0.256
Relative weight ^3^, g/kg BW^2^	2.41 ± 0.13	2.27 ± 0.05	2.10 ± 0.10	2.07 ± 0.10	0.084	0.011	0.036
Intestinal index ^3^, g/cm	0.10 ± 0.01	0.09 ± 0.01	0.09 ± 0.01	0.09 ± 0.01	0.847	0.086	0.092
Ileum							
Weight, g	0.67 ± 0.06	0.63 ± 0.13	0.62 ± 0.05	0.67 ± 0.13	0.874	0.982	0.909
Length, cm	5.36 ± 0.13	5.34 ± 0.30	5.18 ± 0.31	4.62 ± 0.11	0.127	0.032	0.054
Relative weight ^3^, g/kg BW ^2^	0.05 ± 0.01	0.03 ± 0.01	0.05 ± 0.01	0.05 ± 0.01	0.251	0.486	0.477
Intestinal index ^3^, g/cm	0.13 ± 0.01	0.11 ± 0.01	0.12 ± 0.01	0.12 ± 0.01	0.529	0.852	0.344

^1^ T1, T2, T3, and T4 were diets in which the ratios of insoluble to soluble fiber were 3.89, 5.59, 9.12, and 12.81, respectively. ^2^ Lin and quad, linear and quadratic effect of different ratios of insoluble to soluble fiber in pregnant sow diets; BW, body weight. ^3^ The relative weight was calculated as the intestinal weight divided by sow BW, and the intestinal index was calculated as the intestinal weight divided by the intestine length.^4^ Values are mean ± standard error (*n* = 6). ^a,b^ Means with different superscripts within a row differ (*p* < 0.05).

**Table 7:** Effects of the ratio of insoluble to soluble fiber in sow gestation diets on the intestinal development of weaned piglets.

Items ^4^	Treatments ^1^				*p*-Values		
	T1	T2	T3	T4	Treatment	Lin ^2^	Quad ^2^
Duodenum							
Weight, g	3.78 ± 0.45	4.50 ± 0.36	4.86 ± 0.39	4.40 ± 0.64	0.461	0.303	0.273
Length, cm	12.70 ± 1.35	14.78 ± 0.73	13.76 ± 0.30	14.80 ± 2.87	0.772	0.463	0.730
Relative weight ^3^, g/kg BW ^2^	0.50 ± 0.04	0.57 ± 0.03	0.64 ± 0.06	0.57 ± 0.09	0.373	0.263	0.249
Intestinal index ^3^, g/cm	0.30 ± 0.02	0.30 ± 0.02	0.35 ± 0.03	0.32 ± 0.05	0.594	0.294	0.244
Jejunum							
Weight, g	175.70 ± 14.22	203.32 ± 21.16	203.43 ± 8.99	197.74 ± 5.00	0.447	0.287	0.273
Length, cm	783.68 ± 44.08	826.57 ± 22.01	758.28 ± 23.56	794.58 ± 24.44	0.461	0.795	0.962
Relative weight ^3^, g/kg BW ^2^	23.32 ± 1.59	25.59 ± 1.71	26.98 ± 1.08	25.58 ± 1.19	0.356	0.209	0.199
Intestinal index ^3^, g/cm	0.22 ± 0.01	0.25 ± 0.02	0.27 ± 0.01	0.25 ± 0.01	0.214	0.134	0.123
Ileum							
Weight, g	6.32 ± 0.74	7.76 ± 0.75	8.23 ± 0.90	8.15 ± 0.83	0.331	0.104	0174
Length, cm	15.35 ± 1.54	17.22 ± 2.17	17.77 ± 1.07	20.10 ± 1.43	0.248	0.043	0.133
Relative weight ^3^, g/kg BW ^2^	0.84 ± 0.09	0.99 ± 0.09	1.08 ± 0.08	1.06 ± 0.12	0.327	0.096	0.171
Intestinal index ^3^, g/cm	0.42 ± 0.03	0.46 ± 0.03	0.46 ± 0.03	0.41 ± 0.04	0.589	0.931	0.374

^1^ T1, T2, T3, and T4 were diets in which the ratios of insoluble to soluble fiber were 3.89, 5.59, 9.12, and 12.81, respectively. ^2^ Lin and quad, linear and quadratic effect of different ratios of insoluble to soluble fiber in pregnant sow diets; BW, body weight. ^3^ The relative weight was calculated as the intestinal weight divided by sow BW, and the intestinal index was calculated as the intestinal weight divided by the intestine length. ^4^ Values are mean ± standard error (*n* = 6).

**Table 8:** Effects of the ratio of insoluble to soluble fiber in sow gestation diets on the morphometric measurements in the duodenum, jejunum, and ileum in neonatal piglets.

Items ^3^	Treatments ^1^				*p*-Values		
	T1	T2	T3	T4	Treatment	Lin ^2^	Quad ^2^
Duodenum							
Villus height (μm)	0.41 ± 0.03	0.43 ± 0.06	0.46 ± 0.03	0.36 ± 0.04	0.344	0.337	0.227
Crypt depth (μm)	11.62 ± 0.78	12.10 ± 0.64	11.08 ± 0.82	12.48 ± 0.90	0.652	0.590	0.759
Villus/crypt ratio	3.64 ± 0.35 ^ab^	3.56 ± 0.39 ^ab^	4.19 ± 0.40 ^a^	2.81 ± 0.14 ^b^	0.049	0.176	0.089
Jejunum							
Villus height (μm)	0.73 ± 0.05 ^ab^	0.79 ± 0.06 ^a^	0.58 ± 0.05 ^b^	0.60 ± 0.06 ^b^	0.037	0.032	0.101
Crypt depth (μm)	5.96 ± 0.20	6.16 ± 0.41	6.55 ± 0.60	6.52 ± 0.45	0.772	0.313	0.589
Villus/crypt ratio	12.27 ± 0.97 ^a^	12.80 ± 0.33 ^a^	9.01 ± 0.61 ^b^	9.49 ± 1.14 ^b^	0.006	0.006	0.024
Ileum							
Villus height (μm)	0.46 ± 0.07	0.48 ± 0.02	0.45 ± 0.03	0.43 ± 0.08	0.900	0.587	0.767
Crypt depth (μm)	8.53 ± 0.52	10.06 ± 0.70	9.81 ± 1.07	8.10 ± 0.78	0.256	0.683	0.126
Villus/crypt ratio	5.46 ± 0.86	4.87 ± 0.25	4.77 ± 0.39	5.28 ± 0.90	0.866	0.828	0.689

^1^ T1, T2, T3, and T4 were diets in which the ratios of insoluble to soluble fiber were 3.89, 5.59, 9.12, and 12.81, respectively. ^2^ Lin and quad, linear and quadratic effect of different ratios of insoluble to soluble fiber in pregnant sow diets. ^3^ Values are mean ± standard error (*n* = 6). ^a,b^ Means with different superscripts within a row differ (*p* < 0.05).

**Table 9:** Effects of the ratio of insoluble to soluble fiber in sow gestation diets on the morphometric measurements in the duodenum, jejunum, and ileum in weaned piglets.

Items ^3^	Treatments ^1^				*p*-Values		
	T1	T2	T3	T4	Treatment	Lin ^2^	Quad ^2^
Duodenum							
Villus height (μm)	0.35 ± 0.05	0.38 ± 0.04	0.42 ± 0.04	0.35 ± 0.04	0.600	0.761	0.431
Crypt depth (μm)	10.94 ± 1.16	11.49 ± 0.72	11.20 ± 0.60	9.74 ± 0.37	0.533	0.402	0.323
Villus/crypt ratio	3.27 ± 0.53	3.33 ± 0.13	3.76 ± 0.35	3.58 ± 0.42	0.803	0.433	0.682
Jejunum							
Villus height (μm)	0.27 ± 0.07	0.30 ± 0.04	0.28 ± 0.03	0.25 ± 0.02	0.872	0.676	0.711
Crypt depth (μm)	8.39 ± 0.83 ^b^	7.99 ± 1.28 ^b^	13.13 ± 1.40 ^a^	12.53 ± 0.02 ^ab^	0.045	0.017	0.062
Villus/crypt ratio	3.25 ± 0.79	4.02 ± 0.64	2.27 ± 0.41	2.51 ± 0.66	0.261	0.191	0.415
Ileum							
Villus height (μm)	0.25 ± 0.04	0.28 ± 0.04	0.28 ± 0.03	0.31 ± 0.03	0.674	0.213	0.469
Crypt depth (μm)	7.87 ± 0.84	7.86 ± 1.37	8.23 ± 0.59	8.29 ± 0.75	0.973	0.649	0.904
Villus/crypt ratio	3.18 ± 0.22	3.69 ± 0.63	3.45 ± 0.38	3.75 ± 0.17	0.622	0.282	0.559

^1^ T1, T2, T3, and T4 were diets in which the ratios of insoluble to soluble fiber were 3.89, 5.59, 9.12, and 12.81, respectively. ^2^ Lin and quad, linear and quadratic effect of different ratios of insoluble to soluble fiber in pregnant sow diets. ^3^ Values are mean ± standard error (*n* = 6). ^a,b^ Means with different superscripts within a row differ (*p* < 0.05).

**Table 10:** Effects of the ratio of insoluble to soluble fiber in sow gestation diets on enzymatic activity in the jejunum in neonatal and weaned piglets.

Items ^3^	Treatments ^1^				*p*-Values		
	T1	T2	T3	T4	Treatment	Lin ^2^	Quad ^2^
Neonatal piglets, U/mg protein							
Lactase	892.47 ± 136.71 ^a^	724.95 ± 66.95 ^ab^	453.25 ± 75.58 ^bc^	415.88 ± 90.51 ^c^	0.006	0.001	0.005
Sucrase	36.42 ± 8.39 ^a^	33.42 ± 2.54 ^a^	29.39 ± 2.27 ^a^	13.70 ± 1.13 ^b^	0.021	0.004	0.008
Maltase	97.78 ± 19.35	85.98 ± 9.24	58.68 ± 8.67	59.10 ± 6.65	0.076	0.018	0.058
Weaned piglets, U/mg protein							
Lactase	303.54 ± 30.69 ^a^	235.92 ± 21.07 ^a^	257.68 ± 48.42 ^a^	141.41 ± 13.64 ^b^	0.012	0.008	0.027
Sucrase	213.87 ± 34.12 ^a^	182.30 ± 16.57 ^ab^	130.08 ± 14.66 ^bc^	72.90 ± 22.74 ^c^	0.002	<0.001	0.002
Maltase	181.05 ± 28.89 ^a^	154.33 ± 14.03 ^ab^	110.12 ± 12.41 ^bc^	61.72 ± 19.25 ^c^	0.002	<0.001	0.002

^1^ T1, T2, T3, and T4 were diets in which the ratios of insoluble to soluble fiber were 3.89, 5.59, 9.12, and 12.81, respectively. ^2^ Lin and quad, linear and quadratic effect of different ratios of insoluble to soluble fiber in pregnant sow diets. ^3^ Values are mean ± standard error (*n* = 6). ^a–c^ Means with different superscripts within a row differ (*p* < 0.05).
